# Supplementary material for: MultiGWAS: An integrative tool for Genome Wide Association Studies in tetraploid organisms
Source: Ecol Evol. 2021 May 12;11(12):7411–26. doi: 10.1002/ece3.7572 (PMC8216910; doi:10.1002/ece3.7572)
Supplement: Supplementary file 1 — Supplementary Material [file ECE3-11-7411-s001.pdf]

# MultiGWAS: una herramienta de integración para estudios de asociación del genoma completo (GWAS) en organismos tetraploides

L. Garreta[1], I. Cerón-Souza[1], M.R. Palacio[2], and P.H. Reyes-Herrera[1]

[1]Corporación Colombiana de Investigación Agropecuaria (AGROSAVIA), CI Tibaitatá, Kilómetro 14, Vía a Mosquera,7250047, Colombia.

[2]Corporación Colombiana de Investigación Agropecuaria (AGROSAVIA), CI El Mira, Kilómetro 38, Vía Tumaco Pasto, Colombia.

## Resumen

Los estudios de asociación del genoma completo (GWAS) son esenciales para determinar las bases genéticas de la variación fenotípica, ya sea ecológica o económica, entre individuos de poblaciones de organismos modelo y no modelo. Para esta pregunta de investigación, es habitual la replicación de los GWAS probando diferentes parámetros y modelos con el fin de validar la reproducibilidad de los resultados. Sin embargo, todavía faltan metodologías que faciliten la replicación en datos tetraploides de manera directa. Para resolver este problema, diseñamos MultiGWAS, una herramienta que realiza GWAS para organismos diploides y tetraploides ejecutando en paralelo cuatro software, dos diseñados para datos poliploides (GWASpoly y SHEsis) y dos para datos diploides (GAPIT y TASSEL). MultiGWAS tiene varias ventajas. Se ejecuta en la línea de comandos o en una interfaz gráfica; maneja diferentes formatos de genotipos, incluyendo VCF.  Además, permite controlar la estructura de la población, el parentesco y varias medidas de control de calidad de los datos de genotipos. Además, MultiGWAS puede probar modelos de acción génica aditivos y dominantes, y mediante una función de puntuación diseñada en MultiGWAs, seleccionar el mejor modelo para informar de sus asociaciones. Por último, genera varios informes que facilitan la identificación de falsas asociaciones tanto del SNP significativo como del mejor asociado entre los cuatro programas. Probamos MultiGWAS con datos públicos de papas tetraploides para la forma del tubérculo y

varios datos simulados bajo modelos aditivos y dominantes. Estas pruebas demostraron que MultiGWAS es mejor para detectar asociaciones fiables que utilizando cada uno de los cuatro software por separado. Además, el análisis paralelo de los software poliploides y diploides que ofrece MultiGWAS mostró ser muy útil para entender el mejor modelo genético detrás de la asociación de SNP en organismos tetraploides. Por lo tanto, MultiGWAS es una excelente alternativa para hacer la replicación de GWAS en organismos diploides y tetraploides en un único ambiente.

Contacto: phreyes@agrosavia.co

Palabras clave: GWAS en poliploides, GWASPoly, GAPIT, SNP, SHEsis, software, TASSEL

## 1. Introducción

Los estudios de asociación de todo el genoma (GWAS) incluyen pruebas estadísticas que identifican qué variantes a través de todo el genoma de un gran número de individuos se asocian con un rasgo específico (Belgum et al., 2012: Cantor et al., 2011). Esta metodología se comenzó a aplicar en humanos y varias plantas modelo, como el arroz, el maíz y Arabidopsis (Cao et al., 2011; Han & Huang, 2013; Korte & Farlow, 2013; Lauc et al., 2010; Tian et al., 2011). Debido a los avances en la tecnología de secuenciación de alto rendimiento y la disminución del coste de la secuenciación, en los últimos años ha habido un aumento en la disponibilidad de las secuencias del genoma de diferentes organismos a un ritmo más rápido (Ekblom & Galindo, 2011; Ellegre, 2014). Así, el GWAS se está convirtiendo en la herramienta estándar para entender las bases genéticas de la variación fenotípica ecológica o económicamente relevante tanto para los organismos modelo como para los que no lo son. Este incremento incluye especies complejas como los poliploides (Figura1) (Ekblom & Galindo, 2011; Santure & Garant, 2018).

El GWAS aplicado a especies poliploides tiene cuatro retos asociados. En primer lugar, la replicación entre las herramientas es fundamental para validar los resultados del GWAS y capturar las asociaciones positivas (Chanock et al., 2007; De et al., 2014). Debido a que cada herramienta tiene sus propios supuestos (ej., el control de la calidad de los datos, el control de los

falsos positivos, y las optimizaciones del modelo, entre otros), esto genera diferentes resultados tales como los valores p, los umbrales de significación, y los factores de inflación de control genómico. Como consecuencia, cada herramienta puede considerarse como un entorno independiente para replicar un análisis GWAS. Por ejemplo, el umbral de significacia para el valor de p cambia a través de cuatro softwares GWAS (PLINK, TASSEL, GAPIT, y FaST-LMM) cuando el tamaño de la muestra varía (Yan et al., 2019). Esto significa que el listado de SNP con mejor ranking de asociación encontrados en un paquete pueden tener una clasificación diferente en otro.

En segundo lugar, hay muy pocas herramientas centradas en la integración de varios softwares GWAS para comparar diferentes parámetros y condiciones entre ellos. Hasta donde tenemos conocimiento, sólo hay dos softwares con este servicio en mente: iPAT y easyGWAS. El iPAT permite ejecutar en una interfaz gráfica tres softwares de GWAS conocidos que usan línea de comandos como GAPIT, PLINK, y FarmCPU (Zhang et al., 2018). Sin embargo, la salida de cada paquete está separada. Por otro lado, el easyGWAS permite ejecutar un análisis GWAS en la web utilizando diferentes algoritmos y combinando varios resultados GWAS. Este análisis se ejecuta independientemente de la capacidad del ordenador y del sistema operativo. Sin embargo, necesita o bien varios conjuntos de datos para obtener los diferentes resultados de GWAS para hacer réplicas o bien resultados de GWAS ya calculados. En cualquiera de los dos casos, los resultados de los diferentes algoritmos también están separados (Grimm et al., 2017). Por lo tanto, aunque ambos softwares iPAT y easyGWAS se integran con diferentes programas o algoritmos, les hace falta una salida que permita comparar similitudes y diferencias en la asociación.

En tercer lugar, aunque existen diferentes softwares de GWAS para repetir el análisis en diferentes condiciones (Gumpinger et al., 2018), la mayoría de ellos están diseñados exclusivamente para la matriz de datos diploide (Bourke et al., 2018). Por lo tanto, a menudo es necesario “diploidizar” los datos genómicos poliploides para replicar el análisis. Este proceso hace que se ignore en el análisis cómo la dosis alélica afecta a la expresión del fenotipo en las especies poliploides (Ferrao et al., 2018). Sin embargo, se ha observado que algunas secciones del genoma de las especies autopoliploides no se duplican, dando lugar a la herencia disómica de

esos loci (Ohno, 1970, Lynch y Conery, 2000, Dufresne et al., 2014). Además, aún se desconoce el mecanismo de herencia de la mayoría de las especies poliploides. Por lo tanto, un software que tenga en cuenta tanto los datos poliploides como los diploides facilita el análisis de ambos tipos de herencia en los poliploides.

Por último, en el caso de las especies poliploides, cualquier herramienta que integre y compare la acción de diferentes genes entre los softwares es clave para entender cómo la redundancia o la interacción compleja entre alelos afecta a la expresión del fenotipo y a la evolución de nuevos fenotipos (Bourke et al., 2018; Ferrao et al., 2018; Rosyara et al., 2016).

Para superar todos estos desafíos, en este estudio se desarrolló la herramienta MultiGWAS que realiza análisis GWAS para especies diploides y tetraploides utilizando cuatro softwares en paralelo. La herramienta incluye GWASpoly (Rosyara et al., 2016) y SHEsis (Shen et al., 2016) que aceptan datos genómicos poliploides. También incluye GAPIT (Tan et al., 2016) y TASSEL (Bradbury et al., 2007), diseñados para GWAS en plantas, pero que en el caso de datos tetraploides, su uso requiere "diploidizar" la matriz genómica. Esta herramienta trabaja con diferentes formatos de archivos de entrada que provienen de varios softwares de llamada de genotipos poliploides, incluyendo VCF. Además, MultiGWAS hace el preprocesamiento de datos, busca asociaciones ejecutando cuatro softwares GWAS en paralelo, y crea una puntuación para elegir entre los modelos de acción de los genes en GWASPoly y TASSEL. Este estudio describe MultiGWAS y su evaluación a través de estudios de datos simulados y un conjunto de datos públicos de GWAS, demostrando sus ventajas.

## 2. Métodos

La herramienta MultiGWAS consta de tres pasos principales: el ajuste, el multi-análisis y la integración (Figura 2). En el paso de ajuste, MultiGWAS procesa el archivo de configuración. A continuación, limpia y filtra los conjuntos de datos de genotipo y fenotipo, y en el caso de los tetraploides, MultiGWAS "diploidiza" los datos genómicos. A continuación, durante el multi-análisis, cada herramienta GWAS se ejecuta en paralelo. Posteriormente, en el paso de integración, la herramienta MultiGWAS explora los archivos de datos de salida de los cuatro

paquetes (es decir, GWASPoly, SHEsis, GAPIT, y TASSEL); post-procesa los datos y finalmente, genera un resumen de todos los resultados que contienen: tablas de asociaciones, diagramas de Venn mostrando los SNPs asociados compartidos entre las herramientas, SNPs en desequilibrio de ligamiento (LD), diagramas de Manhattan y diagramas cuantil-cuantil (QQ), diagrama de cuerda mostrando la posición en el cromosoma de los SNPs asociados y finalmente perfiles de SNP (ver Sección 2.3.3).

## 2.1. Etapa de ajuste

MultiGWAS toma como entrada un archivo de configuración donde el usuario especifica los datos genómicos y los parámetros de las cuatro herramientas. Una vez leído y procesado el archivo de configuración, los archivos de datos genómicos (genotipo y fenotipo) se limpian, se filtran y se comprueba la calidad de los datos. La salida de esta etapa corresponde a las entradas para los cuatro programas en la etapa de análisis múltiple.

### 2.1.1. Lectura del archivo de configuración.

El archivo de configuración incluye los siguientes ajustes que describimos brevemente:

**Ploidía**: Actualmente, MultiGWAS admite genotipos diploides y tetraploides, donde 2 indica diploides y 4 indica tetraploides.

**Archivos genómicos de entrada:** MultiGWAS utiliza principalmente dos archivos de entrada para el genotipo y el fenotipo. Dependiendo del formato del genotipo (véase más adelante) podría ser necesario un archivo mapa con la información de los marcadores (nombre del cromosoma, la posición genómica, alelo de referencia, y el alelo alternativo).

Para los genotipos alineados con un genoma de referencia, especifique los N cromosomas/contigs mostrados en los gráficos. Los cromosomas están ordenados de forma decreciente por su tamaño. El tamaño del cromosoma/contig se aproxima a la posición de la variante más alta. Cuando los nombres de los cromosomas/contig son numéricos o son

demasiado grandes, se cambian con el prefijo de cadena “contig” y un número secuencial de 1 a N.

MultiGWAS trabaja con datos de genotipos en cinco formatos diferentes: ``gwaspoly" (Rosyara et al., 2016), “vcf” (Team, 2015), “matriz”, “fitpoly” (Voorrips & Gort, 2018), y “updog” (Gerard & Ferrao, 2020). Los dos primeros ya incluyen información de marcadores, pero los tres últimos formatos no, y necesitan el archivo de mapa adicional. Los archivos VCF se transforman en formato GWASpoly utilizando NGSEP 4.0.2 (Tello et al., 2019). Una información detallada de estos archivos y formatos está disponible en el GitHub de la herramienta (https://github.com/agrosavia-bioinfo/MultiGWAS).

**Probando los modelos**: Uno de los principales factores para detectar asociaciones reales entre rasgos y marcadores depende de los modelos de acción génica. Una característica única que ofrece MultiGWAS es la de probar los diferentes modelos de acción génica soportados por las herramientas (ver sección 2.2.). El modelo aditivo es el modelo por defecto aceptado por todas las herramientas. Sin embargo, GWASpoly acepta ocho, TASSEL tres, GAPIT dos, y SHEsis sólo acepta uno.

Para integrar los diferentes modelos en una herramienta envolvente, MultiGWAS ofrece tres opciones de prueba: “aditivo” (aceptado por todas las herramientas), “dominante'” (aceptado por todas las herramientas excepto SHEsis), y “todo'” (para probar todos los efectos aceptados por las herramientas, incluyendo tanto los efectos aditivos como los dominantes). En cualquiera de las tres pruebas, MultiGWAS informa de sus N asociaciones más importantes con un valor bajo de p (Con un N definido por el usuario, véase más abajo). Tomar estas asociaciones es sencillo para las dos primeras pruebas, pero no para la última, ya que las herramientas informan de diferentes asociaciones para cada modelo de acción génica. Para esta última prueba, hemos creado un método que selecciona automáticamente el “mejor” modelo de acción génica descrito en la sección 2.3.1.

**Modelo de GWAS:** MultiGWAS trabaja con fenotipos cuantitativos y ejecuta dos tipos de GWAS, ya sea con control de la estructura de la población y la relación entre las muestras o sin

ningún control. El primero se conoce en la literatura como Q+K o modelo completo, donde Q se refiere a la estructura de la población y K al parentesco; y el segundo se conoce como modelo "naive" (Sharma et al., 2018).

Ambos modelos son enfoques de regresión lineal, y las herramientas GWAS utilizadas por MultiGWAS implementan algunas variaciones de esos modelos. El modelo *naive* se modela con Modelos Lineales Generalizados (GLMs, Fenotipo + Genotipo), y el modelo completo se modela con Modelos Lineales Mixtos (MLMs, Fenotipo + Genotipo + Estructura + Parentesco). El modelo por defecto es utilizado por MultiGWAS es el modelo completo (Q+K) (Yu et al. 2006), siguiendo la ecuación:

$$y = X\beta + S\alpha + Q\nu + Z\mu + e$$

En esta ecuación, el $y$ es el vector de fenotipos observados. Además, $\beta$ es un vector de efectos fijos distintos de los efectos del SNP o del grupo de población, $\alpha$ es un vector de efectos del SNP (Quantitative Trait Nucleotides), el $\nu$ es un vector de efectos de la población, el $\mu$ es un vector de efectos poligénicos de fondo, y el $e$ es un vector de efectos residuales. Además, $Q$ es modelado como un efecto fijo y se refiere a la matriz de incidencia para las covariables de la subpoblación que relacionan $y$ con $\nu$, y $X$, $S$ y $Z$ son matrices de incidencia de 1s y 0s que relacionan $y$ con $\beta$, $\alpha$ y $\mu$, respectivamente.

**Significancia de todo el genoma**: El GWAS busca SNPs asociados con el fenotipo de forma estadísticamente significativa. Se especifica un umbral o nivel de significancia α y se compara con el valor p derivado de cada puntaje de la asociación. Los niveles de significancia estándar son 0,01 o 0,05 (Gumpinger et al., 2018, Rosyara et al., 2016), y MultiGWAS utiliza un α de 0,05 para los cuatro paquetes GWAS. Sin embargo, en GWASpoly y TASSEL, que calcula el efecto del SNP para cada clase genotípica utilizando diferentes modelos de acción génica (véase "Etapa de análisis múltiple"), el umbral se ajusta según estos dos paquetes. Por lo tanto, el número de marcadores analizados puede ser diferente en cada modelo (véase más adelante), lo que repercute en los umbrales de los valores p.

**Corrección de pruebas múltiples**: Debido al enorme número de pruebas estadísticas realizadas por los GWAS, es necesario realizar un método de corrección para las pruebas de hipótesis múltiples y ajustar el umbral del valor p de manera acorde. Dos métodos estándar para las pruebas de hipótesis múltiples son la tasa de falsos descubrimientos (FDR) y la corrección de Bonferroni. Este último es el método por defecto utilizado por MultiGWAS, que es uno de los métodos más rigurosos. Sin embargo, en lugar de ajustar los valores p, MultiGWAS ajusta el umbral por debajo del cual un valor p se considera significativo. Es decir, $\alpha/m$, donde $\alpha$ es el nivel de significancia, y m es el número de marcadores probados de la matriz de genotipos.

**Número de asociaciones notificadas**: El uso de niveles estrictos de significancia puede descartar muchas asociaciones con valores p más cercanos al umbral de significación, generando un elevado número de falsos negativos (Thompson et al., 2011, Kaler y Purcell, 2019).
Para evitar este problema, MultiGWAS ofrece la opción de especificar el número de asociaciones mejor clasificadas (valores p más bajos), añadiendo el valor p correspondiente a cada asociación encontrada. De esta manera, es posible ampliar el número de resultados y su replicabilidad a través de los diferentes programas. No obstante, el informe muestra cada asociación con su correspondiente valor p.

**Filtros de control de calidad**: Un paso de control es necesario para revisar errores o baja calidad de los datos de entrada para el genotipo o fenotipo, porque puede resultar en resultados GWAS espurios. MultiGWAS ofrece la opción de seleccionar y definir umbrales para los siguientes filtros que controlan la calidad de los datos: Frecuencia del alelo Menor (MAF), tasa de individuos perdidos (MIND), tasa de SNP perdidos (GENO) y umbral de Hardy- Weinberg (HWE). Todos estos filtros son implementaciones integradas de multiGWAS, excepto el HWE para tetraploides:
- MAF de x: filtra los SNPs con frecuencia alélica menor por debajo de x (por defecto 0,01);
- MIND de x: filtra todos los individuos con genotipos perdidos superiores a x*100% (por defecto 0,1);
- GENO de x: filtra los SNPs con valores faltantes que superan el x*100% (por defecto 0,1);

- HWE de x: filtra los SNP con un valor p inferior al umbral de x en la prueba exacta de equilibrio de Hardy-Weinberg. En el caso de los genotipos tetraploides este cálculo se toma de SHEsis [Shen et al., 2016].

**Herramientas GWAS**: Lista de los cuatro nombres de software GWAS para ejecutar e integrar en el análisis MultiGWAS: GWASpoly y SHEsis (diseñados para datos poliploides), y GAPIT y TASSEL (diseñados para datos diploides).

**Umbral de desequilibrio de enlaces ($R^2$)**: Umbral de correlación al cuadrado ($R^2$) definido por el usuario por encima del cual se considera que un par de SNP está en desequilibrio de ligamiento (véase la sección 2.3.3 para más detalles).

### 2.1.2. Preprocesamiento de los datos

Una vez procesado el archivo de configuración, los datos genómicos se leen y se limpian seleccionando los individuos presentes tanto en el genotipo como en el fenotipo. A continuación, MultiGWAS elimina los individuos y SNPs de baja calidad siguiendo los filtros de control de calidad seleccionados anteriormente y sus umbrales. En este punto, el formato "ACGT" adecuado para el software poliploide GWASpoly y SHEsis, se "diploidiza" para GAPIT y TASSEL. Los genotipos tetraploides homocigóticos se convierten en diploides de esta manera: AAAA→AA, CCCC→CC, GGGG→GG, TTTT→TT. Además, para los genotipos heterocigotos tetraploides, la conversión depende de los alelos de referencia y alternativos calculados para cada posición (por ejemplo, AAAT→AT, ... ,CCCG→CG).
Después de este proceso, MultiGWAS convierte los datos genómicos, el genotipo y los conjuntos de datos del fenotipo a los formatos específicos requeridos para cada uno de los cuatro paquetes GWAS.

## 2.2.Estado de análisis múltiple

Como se describe en la sección 2.1.1, MultiGWAS puede ejecutar dos tipos de GWAS: naive sin ningún control de datos de genotipo y full con control de la estructura de la población y el

parentesco. GWASpoly, GAPIT y TASSEL admiten ambos modelos. Sin embargo, SHEsis sólo admite el modelo naive. Para controlar la estructura de la población y el parentesco en el modelo completo, MultiGWAS utiliza algoritmos incorporados para calcular los componentes principales como covariables y el parentesco entre pares de individuos. A continuación, se describe con más detalle cada una de las herramientas GWAS.

### 2.2.1 GWASpoly:

GWASpoly (Rosyara et al., 2016) es un paquete de R diseñado para GWAS en especies poliploides utilizado en varios estudios en plantas (Berdugo-Cely et al., 2017, Ferrão et al., 2018, Sharma et al., 2018, Yuan et al., 2019). GWASpoly utiliza un modelo lineal mixto Q+K con SNPs bialélicos que tienen en cuenta la estructura de la población y el parentesco. Además, para calcular el effecto de SNP para cada clase genotípica, GWASpoly proporciona ocho modelos de acción génica: general, aditivo, alterno dominante simplex, referencia dominante simplex, alternativo dominante dúplex, dominante dúplex, diplo general y diplo aditivo. En consecuencia, el número de pruebas estadísticas realizadas puede ser diferente en cada modelo de acción, y por tanto los umbrales por debajo de los cuales los valores p se consideran significantes.

MultiGWAS utiliza la versión 1.3 de GWASpoly con todos los modelos de acción de genes disponibles para buscar asociaciones. El MultiGWAS reporta los N mejores rankeados (los SNPs con los valores p más bajos) que el usuario especificó en el archivo de configuración de entrada N. El modelo completo utilizado por GWASpoly incluye la estructura de la población y el parentesco, que se estiman utilizando los primeros cinco componentes principales y la matriz de parentesco, respectivamente, ambos calculados con los algoritmos incorporados de GWASpoly. En los algoritmos incorporados.

### 2.2.2 SHEsis:

SHEsis (Shen et al., 2016) es un programa basado en un modelo de regresión lineal que incluye análisis de asociación de un solo locus para poliploides, entre otros análisis. Sin embargo, ha sido

utilizado principalmente por animales y humanos, ambos diploides (Qiao et al., 2015, Meng et al., 2019).

MultiGWAS utiliza la versión 1.0, que no tiene en cuenta la estructura de la población ni el parentesco. Sin embargo, MultiGWAS estima externamente el parentesco para SHEsis excluyendo a los individuos con parentesco de primer grado críptico utilizando la matriz de parentesco calculada por el algoritmo incorporado GWASpoly.

### 2.2.3 GAPIT:

GAPIT es un programa basado en R diseñado para plantas. Esta herramienta implementa el MLM clásico para el modelo completo, corrigiendo por la estructura de la población y la relatividad. Además, utiliza el enfoque GLM para el modelo ingenuo sin ninguna corrección (Tang et al., 2016).

GAPIT ofrece dos modelos de acción génica: aditivo y dominante. Para ambos modelos, el genotipo debe estar en formato numérico. Para el modelo aditivo, el genotipo es transformado implícitamente por GAPIT, utilizando 0 para genotipos homocigotos con combinaciones de alelos recesivos, 2 para genotipos homocigotos con combinaciones de alelos dominantes y 1 para genotipos heterocigotos. Para el modelo dominante, MultiGWAS transforma el genotipo, utilizando 0 para los dos tipos de genotipos homocigotos y 1 para los genotipos heterocigotos, como indican los autores (Tang et al., 2016). MultiGWAS utiliza la última versión 3, que también implementa varios métodos de vanguardia desarrollados para la genómica estadística (Wang y Zhang, 2020).

### 2.2.4 TASSEL:

TASSEL es otro programa estándar de GWAS desarrollado inicialmente para el maíz pero que actualmente se utiliza en varias especies [Álvarez et al., 2017, Zhang et al., 2018]. TASSEL es un paquete Java que se ejecuta mediante una interfaz gráfica de usuario desarrollada en JAVA o

una interfaz de línea de comandos a través de un pipeline de Perl. En MultiGWAS se implementa el pipeline de Perl.

Para el análisis de asociación, TASSEL incluye el modelo lineal general (GLM) para un análisis ingenuo. Además, utiliza el modelo lineal mixto (MLM) para un análisis completo controlando la estructura de la población, un análisis de componentes principales, y controlando el parentesco utilizando una matriz de parentesco con un método IBS centrado con algoritmos incorporados en TASSEL. Además, como GWASPoly, TASSEL proporciona modelos de acción de tres genes para calcular el efecto de cada clase genotípica de SNP: general, aditivo y dominante. Por lo tanto, el umbral de significancia depende de cada modelo de acción.

### 2.3 Etapa de integración.

Los resultados de los cuatro paquetes de GWAS se procesan posteriormente para identificar los SNP con valores p significativos de asociación o con la mejor asociación (es decir, con valores p cercanos a un umbral de significancia).

#### 2.3.1 Selección del mejor modelo de acción génica

MultiGWAS ofrece tres opciones de análisis: "aditivo", "dominante" y "todos". Tomar las mejores asociaciones de las pruebas "aditivas" y "dominantes" es sencillo. Sin embargo, para la opción "todos", MultiGWAS tiene un método para seleccionar dentro de cada herramienta el "mejor" modelo de acción génica y tomar las mejores asociaciones.

El método funciona puntuando cada modelo de acción génica utilizando tres criterios: factor de inflación (I), SNPs compartidos (R) y SNPs significantes (S), utilizando la siguiente ecuación:

$$score\ (M_i) = I_i + R_i + S_i$$

donde score ($M_i$) es la puntuación para el modelo de acción génica $M_i$, con $i$ de 1..k, para un paquete GWAS con $k$ modelos de acción génica. $I_i$ es la puntuación del factor de inflación

definido como $I_i$ = 1 -|1 -λ($M_i$)|, donde λ($M_i$) es el factor de inflación para el modelo $M_i$. El $R_i$ es el puntaje de los SNP compartidos definido como $R_i = \sum_{j=1}^{k} |M_i \sim M_j|$, donde $|M_i \sim M_j|$ es el número de SNPs compartidos entre los modelos $M_i$ y $M_j$, normalizado por el número máximo de SNPs compartidos entre todos los modelos. Y $S_i$ es el número de SNPs significantes del modelo $M_i$ normalizado por el número total de SNPs compartidos entre todos los modelos.

La puntuación es alta cuando un modelo $M_i$ tiene un factor de inflación λ cercano a 1, identifica un alto número de SNPs compartidos, y contiene uno o más SNPs significantes. Por el contrario, la puntuación es baja cuando el modelo $M_i$ tiene un factor de inflación λ bajo (cercano a 0) o alto (λ > 2), que identifica un pequeño número de SNPs compartidos, y contiene 0 o pocos SNPs significativos. En cualquier otro caso, la puntuación resulta del equilibrio entre el factor de inflación, el número de SNPs compartidos y el número de SNPs significantes.

### 2.3.2 Selección de las asociaciones significativas y mejor clasificadas

MultiGWAS informa de dos grupos de asociaciones de los cuatro paquetes GWAS: las asociaciones estadísticamente significantes con valores p por debajo de un umbral de significancia, y las asociaciones mejor clasificadas con los valores p más bajos, pero que no alcanzan el límite para ser estadísticamente significantes. Sin embargo, representan asociaciones interesantes para su posterior análisis (posibles falsos negativos).

### 2.3.3 Integración de los resultados

Los cuatro paquetes de GWAS adoptados por MultiGWAS utilizan enfoques de regresión lineal. Sin embargo, a menudo producen resultados de asociación diferentes para los mismos datos de entrada. Los valores p calculados para el mismo conjunto de SNPs son diferentes entre paquetes. Por lo tanto, los SNP con valores p significativos para un paquete pueden no ser significativos para los demás. Por otra parte, los SNP bien clasificados en un paquete pueden estar clasificados de forma diferente en otro.

MultiGWAS integra los resultados de las cuatro herramientas generando seis tipos de resultados que combinan gráficos y tablas para comparar, seleccionar e interpretar el conjunto de posibles SNPs asociados a un rasgo de interés (Fig. 3). La salida unificada es un documento HTML que contiene las tablas y los gráficos para cubrir todas las necesidades del usuario para presentar los resultados e incluye:

**Gráficos QQ para asociaciones GWAS**: El gráfico QQ muestra qué tan bien los SNPs se ajustan a la hipótesis nula de no asociación con el fenotipo. Ambas distribuciones deberían coincidir, y la mayoría de los SNP deberían situarse en la línea diagonal roja. Las desviaciones de muchos SNP pueden reflejar valores p inflados debido a la estructura de la población o al parentesco críptico. No obstante, pocos SNP se desvían de la diagonal para un rasgo verdaderamente poligenético (Power et al., 2016). MultiGWAS añade en la parte superior de cada gráfico QQ el correspondiente factor de inflación λ para evaluar el grado de inflación del estadístico de prueba.

**Diagrama de Manhattan para las asociaciones GWAS**: MultiGWAS utiliza los gráficos clásicos de Manhattan para visualizar los resultados de cada paquete. En ambos gráficos, los puntos son los SNPs y sus valores p se transforman en puntuaciones como -log10(valor p) (véase la figura 3). El gráfico de Manhattan muestra la fuerza de asociación de los SNP (eje y) distribuidos en su ubicación genómica (eje x), de modo que cuanto más alta sea la puntuación, más fuerte será la asociación. MultiGWAS añade marcas distintivas al gráfico; los SNP significativos están por encima de una línea roja, los SNP mejor clasificados están por encima de una línea azul, y los SNP compartidos entre paquetes están coloreados en verde.

**Tablas y diagramas de Venn para SNPs individuales y compartidos**: MultiGWAS proporciona Tablas y gráficas para informar de los SNPs mejor clasificados y significativos identificados por los cuatro paquetes GWAS de forma integradora (Figura 3). Tanto los valores p como los niveles de significancia se han escalado como log10(valores p) para dar puntuaciones altas a los SNPs mejor evaluados estadísticamente.

En primer lugar, los SNP mejor valorados corresponden a los N SNP con mayor puntuación, independientemente de que hayan sido evaluados como significantes o no por su paquete, y con N definido por el usuario en el archivo de configuración. Estos SNPs aparecen tanto en una tabla de SNPs como en un diagrama de Venn. La tabla los enumera por paquete y los ordena por puntuación decreciente, mientras que el diagrama de Venn destaca si fueron los mejor clasificados en un solo paquete o en varios a la vez (compartidos). En segundo lugar, los SNP significativos corresponden a los valorados como estadísticamente significativos por cada paquete. También aparecen en un diagrama de Venn y en la tabla de SNPs, marcados con significancia TRUE (T).

**Visualización de los SNPs en desequilibrio de enlace (LD)**: MultiGWAS muestra en un diagrama de Venn y en una tabla (Figuras 7.a y 7.b, respectivamente) los pares de SNPs con correlación al cuadrado igual o mayor que el umbral $R^2$, donde $R^2$ es definido por el usuario en el archivo de configuración (véase 2.1.1). MultiGWAS une las N mejores asociaciones encontradas para cada paquete GWAS (SNPs con el valor p más bajo), calcula para cada par de SNPs el $R^2$ utilizando la librería ldsep de R para LD en poliploides [Gerard, 2021]. Finalmente, resume los resultados en una tabla con pares de SNPs por fila junto con su $R^2$ calculado.

A los pares de SNPs en LD se les asigna un nuevo ID (LD_SNP) y se visualiza en un diagrama de Venn que destaca los SNPs compartidos en LD detectados entre el software GWAS. Esta vista permite una rápida identificación de SNPs relacionados con nombres diferentes en lugar de una tabla simple, como la mayoría de los paquetes GWAS muestran sus resultados.

**Mapas de calor para la estructura de los SNP compartidos**: Para cada SNP identificado más de una vez, MultiGWAS proporciona un perfil del SNP. Se trata de un mapa de calor bidimensional que representa el SNP y que visualiza cada rasgo por individuos y genotipos como filas y columnas, respectivamente.

Dentro de la figura, a la izquierda, los individuos se agrupan en un dendrograma por su genotipo. A la derecha, aparece el nombre o ID de cada individuo. En la parte inferior, los genotipos están ordenados de izquierda a derecha, empezando por el alelo mayor al menor (es decir, AAAA,

AAAB, AABB, ABBB, BBBB). En la parte superior, hay una descripción del rasgo basada en un histograma de frecuencia (arriba a la izquierda) y un color asignado para cada valor numérico del fenotipo utilizando una escala de colores (arriba a la derecha). Así, cada individuo aparece como una línea coloreada por su valor de fenotipo en su columna de genotipo. Para cada columna, hay una línea sólida aguamarina con cada columna de la media y una línea aguamarina punteada que indica cuánto se está desviando la celda de la media (Figura 3).

Como cada reporte de multiGWAS muestra un rasgo específico a la vez, el histograma y el color seguirán siendo los mismos para todos los SNP mejor clasificados.

**Diagramas de cuerda por cromosoma para los SNP**: Los diagramas de cuerda visualizan la ubicación en el genoma de los SNP asociados mejor clasificados que son compartidos entre los cuatro paquetes y descritos en las tablas. Esta visualización complementa los gráficos Manhattan de cada paquete GWAS (Figura 3).

## 3 Disponibilidad e implementación

MultiGWAS es una herramienta de integración desarrollada en R (R>=3.6). Sin embargo, no es un paquete de R ni se ejecuta en la interfaz de R. En su lugar, se ejecuta en entornos Linux porque integra cuatro softwares GWAS externos implementados en diferentes lenguajes. GWASpoly y GAPIT son paquetes R; SHEsis es un programa binario desarrollado en C++, y TASSEL es un paquete Java que se ejecuta a través de un pipeline implementado en Perl. Por consiguiente, los usuarios pueden ejecutar MultiGWAS mediante una interfaz de línea de comandos (un script R) o una interfaz gráfica de usuario (una aplicación Java). Para obtener instrucciones detalladas y ejemplos de uso, consulte https://github.com/agrosavia-bioinfo/MultiGWAS#running-the-examples.

### 3.1 Parámetros de entrada

MutiGWAS utiliza un único archivo de texto de configuración con los valores de los principales parámetros que dirigen el análisis. Si los usuarios prefieren un archivo de texto, éste debe tener los nombres y valores de los parámetros separados por dos puntos, los nombres de los parámetros sin espacios en blanco, los valores TRUE o FALSE para indicar si se aplican los filtros y el valor NULL para indicar que no hay valor para el parámetro. Este archivo debe tener la estructura mostrada en la Figura 4. En cambio, si los usuarios prefieren la aplicación GUI, pueden crear el archivo de configuración utilizando la GUI descrita en la sección 3.2.2.
Los archivos de entrada (genotipo/fenotipo/mapa) no necesitan estar en el directorio de trabajo, pero si este es el caso, MultiGWAS necesita la ruta absoluta.

### 3.2 Instalación y uso de MultiGWAS

MultiGWAS ofrece diferentes opciones de instalación: desde cero, versiones pre-compiladas, una máquina virtual y una imagen docker. Las instrucciones específicas para los diferentes tipos de instalación, incluyendo una máquina virtual (VM) de Linux lista para usar para ejecutar MultiGWAS en otras plataformas (Windows, OS X), están disponibles en el Github de la herramienta (https://github.com/agrosavia-bioinfo/MultiGWAS).

#### 3.2.1 Uso de la interfaz de línea de comandos

La ejecución de la herramienta CLI es sencilla. En una consola de Linux, se debe ir a la carpeta donde se encuentra el archivo de configuración, y escribir el nombre de la herramienta ejecutable, seguido del nombre del archivo de configuración, así:

```
multiGWAS Test01.config
```

A continuación, la herramienta inicia la ejecución, mostrando información sobre el proceso en la ventana de la consola. Cuando termina, los resultados se encuentran en una nueva subcarpeta llamada “out-Test01”, que contiene una subcarpeta para cada rasgo en el archivo de fenotipo.

Los resultados de cada subcarpeta de un rasgo fenotípico subcarpeta incluye un informe HTML completo que contiene las diferentes vistas descritas en la sección de métodos, los gráficos y tablas de origen que apoyan el informe, y las tablas pre-procesadas de los resultados generados por los cuatro paquetes GWAS utilizados por MultiGWAS.

### 3.2.2 Utilización de la interfaz gráfica para el usuario

La interfaz permite a los usuarios guardar, cargar o especificar los diferentes parámetros de entrada para MultiGWAS de forma amigable (Figura 5). Los parámetros de entrada se corresponden con los ajustes incluidos en el archivo de configuración descrito en la subsección 2.1.1. Se ejecuta llamando al siguiente comando desde una consola Linux:

```
jmultiGWAS
```

## 4 Probando MultiGWAS

### 4.1 Probando MultiGWAS en datos reales

Probamos MultiGWAS en datos reales utilizando un conjunto de datos abierto de un panel de diversidad de información de fenotipo y genotipo para papa tetraploide. Estos datos forman parte del proyecto SOLCAP del USDA-NIFA (Hirsch et al., 2013). Limitamos el experimento sólo al rasgo de la forma del tubérculo, probando tanto el modelo GWAS completo como el naive.

### 4.2 Probando MultiGWAS en datos simulados

Creamos dos conjuntos de datos simulados de genotipo-fenotipo diferentes como experimento para determinar las ventajas de ejecutar una herramienta integradora como MultiGWAS en comparación con un análisis individual de cada uno de los cuatro softwares GWAS que integran MultiGWAS (es decir, GWASpoly, SHEsis, GAPIT y TASSEL). El primer conjunto de datos

simulado se basó en un modelo de herencia aditivo, y el segundo en un modelo de herencia dominante.

En ambas simulaciones, utilizamos como población fundadora un subconjunto de 400 SNP y 150 individuos de los datos de papa tetraploide descritos por Enciso-Rodríguez et al. (2018). Para crear ambas simulaciones de fenotipos, tomamos muestras aditivas o dominantes de una distribución gamma Γ (forma = 0,2 y escala = 5) y especificamos diez SNPs como causales junto con sus efectos bajo el software Phyton3 SeqBreed (Pérez-Enciso et al., 2020), inspirado en el software pSBVB creado para generar datos poliploides (Zingaretti et al., 2019]).

Ambos conjuntos de datos simulados fueron analizados en MultiGWAS utilizando los siguientes parámetros: Acción génica, ya sea aditiva o dominante, filtro de datos falso, método de corrección Bonferroni, y modelo Naive de GWAS utilizando el genotipo fundador y el fenotipo aditivo o dominante. Tras el análisis MultiGWAS, resumimos los principales SNP (es decir, el número N de SNP mejor clasificados encontrados por la herramienta) y los SNP significativos encontrados por cada herramienta GWAS. Posteriormente, calculamos dos métricas: las tasas de verdaderos positivos (TPR) y las tasas de verdaderos negativos (TNR) expresadas en las siguientes ecuaciones:

$$TPR = TP/TP + FN$$

Donde TP es el número de SNPs correctamente identificados como SNPs causales, y FN es el número de SNPs incorrectamente identificados como SNPs no causales.

$$TNR = TN/TN + FP$$

Donde TN es el número de SNPs correctamente identificados como SNPs no causales, y FP es el número de SNPs incorrectamente identificados como SNPs causales.

## 5 Resultados

### 5.1 Desempeño de MultiGWAS en datos reales

Utilizamos MultiGWAS para analizar la forma del tubérculo del conjunto de datos de papa tetraploide utilizando un modelo GWAS completo que controla la estructura de la población y el parentesco (Hirsch et al., 2013).

El análisis GWAS completo encontró varios SNPs asociados (tabla de la Figura 6.a). De ellos, se detectaron tres SNPs denominados c2_25471, c2_45606 y c2_45611, de los principales SNPs en los cuatro paquetes de GWAS (intersección central en la Figura 6.a). Dos SNP, denominados c1_8019 y c2_25471, fueron identificados como significantes por los paquetes GWASpoly y SHEsis (Figura 6.b). Estudios previos de asociación también encontraron asociación en estos mismos SNP, en los que el SNP c1_8019 se asocia con los rasgos de forma del tubérculo de la papa y profundidad del ojo (Rosyara et al., 2016, Sharma et al., 2018), mientras que los SNP c2_45606 y c2_45611 se asocian a la profundidad del ojo (Totsky et al., 2020).

MultiGWAS reforzó la replicabilidad de estos SNPs asociados por los cuatro paquetes GWAS. Además, el análisis de desequilibrio de ligamiento confirmó esta replicabilidad. Asímismo, cuando se utilizó el modelo Naive GWAS para analizar el mismo conjunto de datos, MultiGWAS mostró que las cuatro herramientas detectaron simultáneamente el SNP c1_8019 como un SNP asociado significativo, destacándolo como una asociación confiable. (véase el material suplementario S1 y S2 en https://github.com/agrosaviabioinfo/multiGWAS/tree/master/docs).

Dos pares de SNPs resultaron en LD, c2_8019 con c2_25471 y c2_45606 con c2_45611, denominados por MultiGWAS como LD_SNP1 y LD_SNP2, respectivamente (tabla de la Figura 7.a). El diagrama de Venn (Figura 7.b) muestra que mínimo un SNP de los SNPs emparejados en LD fue detectado por los cuatro paquetes GWAS, mostrando la replicabilidad de los SNPs en los cuatro paquetes. Además, el diagrama de cuerdas muestra que la mayoría de los SNP mejor clasificados estaban en el cromosoma 10 (Figura 7.c). Por último, los mapas de calor de los SNP

mejor clasificados muestran diferencias visibles que relacionan la asociación del genotipo con el fenotipo de la forma del tubérculo (Figura 7.d).

El diagrama de Manhattan para cada paquete GWAS mostró que los cuatro paquetes encontraron que la ubicación de los SNP asociados (es decir, SNP por encima de la línea azul) era el cromosoma 10 (Figura 8). De ellos, para GWASpoly y SHEsis son significantes (SNPs por encima de la línea roja). Ambos grupos de SNP, los fuertemente asociados y los significantes, están presentes tanto en la tabla compartida como en el diagrama de Venn (Figura 6).

Por otro lado, para la mayoría de los paquetes GWAS, excepto para SHEsis, la mayoría de los valores p observados correspondían a los valores p esperados, como se muestra en la Gráficos QQ generados a partir de las asociaciones encontradas para cada paquete (gráficos QQ por encima de los gráficos Manhattan en la Figura 8). En el caso de SHEsis, su factor de inflación genómica $\lambda$ fue muy superior a 1,0, lo que significa que sus puntuaciones calculadas estaban infladas, y se explica porque SHEsis no controla la estructura de la población ni el parentesco.

### 5.2 Desempeño de MultiGWAS en datos simulados

En el caso de MultiGWAS, presentamos los resultados utilizando diferentes conjuntos para evidenciar el efecto de la replicabilidad en el desempeño (MultiGWAS_1: predicho por un software, MultiGWAS_2: predicho por dos softwares, MultiGWAS_3: predicho por tres softwares, y MultiGWAS_4: predicho por cuatro softwares).

Para la simulación del efecto aditivo, GWASpoly (verde) y SHEsis (azul) tuvieron el mejor rendimiento basado en la tasa de verdaderos positivos (TPR) y la tasa de verdaderos negativos (TNR) en la detección del SNP mejor clasificados. Los dos softwares diploides GAPIT y TASSEL, tienen resultados similares pero un rendimiento inferior en ambas estadísticas. Paralelamente, el rendimiento de MultiGWAS cambia en función del número de softwares implicados en la intersección de SNP predichos; el TPR fue progresivamente más bajo, y el TNR progresivamente más alto. En consecuencia, en los dos casos más restrictivos (es decir, la intersección del SNP predicho por tres y los cuatro softwares, MultiGWAS_3 y MultiGWAS_4

respectivamente), el TPR fue similar al obtenido por TASSEL y GAPIT. Sin embargo, el TNR fue mayor que incluso GWASpoly y SHEsis (Figura 9.a).

Para la simulación del efecto dominante, GAPIT (aguamarina) tiende a tener un TPR más alto que los otros tres softwares. Además, SHEsis tuvo un valor más bajo tanto de TPR como de TNR ya que fue diseñado sólo para detectar asociaciones con efectos aditivos. Comparando el rendimiento de estos cuatro softwares con una herramienta de integración como MultiGWAs, esta herramienta tuvo un rendimiento similar a la simulación de efectos aditivos. A medida que la intersección es más restrictiva, el TPR fue progresivamente menor y el TNR progresivamente mayor. Sin embargo, en los dos casos más restrictivos (es decir, la intersección de SNP predichos por tres y los cuatro softwares, MultiGWAS_3 y MultiGWAS_4 respectivamente), el TNR fue mayor que los cuatro softwares (Figura 9.b).

En el caso de los SNP significantes, para los efectos aditivos, SHEsis (azul) obtuvo el TPR más alto pero también el TNR más bajo, lo que sugiere que SHEsis probablemente está sobreestimando la asociación de valores p significativos. Por lo tanto, las asociaciones verdaderas y falsas están alcanzando el umbral de significancia. En comparación, MultiGWAS_4, GWASpoly y GAPIT son más conservadores, con un TPR más cercano pero un TNR alto (Figura 10.a).

Para la simulación de efectos dominantes, GWASpoly tuvo el TPR más alto con un TNR más bajo. Así, este software fue el más sensible detectando asociaciones significativas, pero al mismo tiempo, fue uno de los menos específicos. En comparación, MultiGWAS_4 y GAPIT tuvieron un TPR ligeramente inferior al de GWASpoly, pero con el TNR más alto. Este patrón sugiere que ambos son menos sensibles a la hora de detectar una asociación significativa, pero más específicos que GWASpoly. Por tanto, MultiGWAS_4 proporciona asociaciones muy precisas (figura 10.b).

## 6 Discusión

El reanálisis de los datos de papa y de los datos simulados con MultiGWAS demostró que esta herramienta de integración es útil para mejorar los GWAS de los efectos de acción génica aditiva y dominante en especies diploides y tetraploides. A través del desempeño de MultiGWAS, pudimos comprobar su efectividad para responder a algunos de los retos del análisis de organismos poliploides. Entre ellos se encuentran la integración y replicación entre parámetros y software, la diploidización de datos poliploides y la incorporación de diferentes mecanismos de herencia (Dufresne et al., 2014).

La principal ventaja de MultiGWAS es que replica el análisis GWAS entre cuatro programas informáticos e integra los resultados obtenidos a través de los programas informáticos, los modelos y los parámetros. Por lo tanto, en MultiGWAS, los usuarios no tienen que elegir entre especificidad o sensibilidad porque pueden observar su efecto en el análisis dentro del mismo entorno integrador.

Otra dificultad para la replicación entre softwares es la variabilidad de los formatos de los datos genómicos de entrada. MultiGWAS recibe los datos de genotipo en cinco formatos diferentes, incluyendo dos salidas de software utilizadas para llamar a la dosis de alelos poliploides. Actualmente, el formato más común para los datos de variantes de secuenciación de próxima generación es el VCF (Variant Call Format) (Danecek et al., 2011, Ebbert et al., 2014). Una de las ventajas del VCF es su versatilidad a la hora de resumir información importante del genoma para cientos o miles de individuos y SNP, incluyendo información sobre los niveles de ploidía. MultiGWAS simplifica el uso del software GWAS porque permite los archivos VCF como entrada (pero véase la herramienta VarStats en VTC).

Además, MultiGWAS es la única herramienta de integración que conocemos que facilita la comprensión directa del efecto de diploidizar los datos tetraploides en el ejercicio del análisis. El perfil del SNP permite identificar cuáles son las asociaciones significativas detectadas por más de un software. Además, aunque MultiGWAS inspecciona los SNP significativos basándose en el valor p, es esencial volver a los datos y comprobar si el SNP es una verdadera asociación entre

el genotipo y el fenotipo. Para ello, el perfil del SNP ofrece una información visual sobre la exactitud de la asociación.

Además, el MultiGWAS permite comparar entre los modelos de acción génica que proporciona GWASPoly y TASSEL. GWASpoly (Rosyara et al., 2016 propone modelos de acción génica poliploides, incluyendo aditivos, aditivos diploides, dúplex dominantes, simples y generales. Por otro lado, TASSEL (Bradbury et al., 2007) también modela diferentes tipos de acción génica para diploides generales, aditivos y dominantes. Para elegir entre los modelos, proponemos una selección automática del modelo de acción génica para ambas herramientas basada en un equilibrio entre tres criterios: el factor de inflación, la replicabilidad de los SNP identificados y la significancia de los SNP identificados. Este índice de inflación es una nueva herramienta de comparación que no ofrece ni GWASPoly ni TASSEL. Esta estrategia automática ayudará a comprender el modelo de acción génica para el rasgo de interés. Aunque el foco principal está en los SNPs resultantes, el modelo tiene supuestos que reflejan las acciones génicas de un fenotipo específico.

Finalmente, MultiGWAS, a través de la comparación activa entre modelos, se enfoca en la búsqueda de los mecanismos de herencia comparando entre dos softwares diseñados para la herencia polisómica (Rosyara et al., 2016, Shen et al., 2016) con dos softwares para la herencia disómica (Purcell et al., 2007, Bradbury et al., 2007). Es una pregunta abierta entender los mecanismos de herencia de los organismos poliploides. Para los autopoliploides, la mayoría de los loci tienen una herencia polisómica. Sin embargo, las secciones del genoma que no se duplicaron dan lugar a una herencia disómica para algunos loci (Ohno, 1970, Lynch y Conery, 2000, Dufresne et al., 2014). Por tanto, MultiGWAS es una herramienta valiosa para los investigadores porque busca asociaciones significativas que implican ambos tipos de herencia.

## 6.1 Consideraciones futuras

La evolución y la genómica poblacional de los poliploides es un área de investigación nueva y apasionante. El avance de las técnicas de secuenciación de nueva generación produce cada vez

más datos empíricos de poliploides en diferentes organismos modelo y no modelo (Ekblom y Galindo, 2011, Ellegren, 2014).

Muchos de los supuestos desarrollados para los diploides en el análisis de GWAS no se aplican del todo a los poliploides (Dufresne et al., 2014). Afortunadamente, en los últimos cinco años, se están probando y desarrollando en datos simulados y empíricos diferentes modelos para calcular varios parámetros de genómica poblacional en poliploides (Meirmans et al., 2018, Hardy, 2016, Blischak et al., 2016).

En MutiGWAS, comenzamos con la ploidía más simple, como los tetraploides. Sin embargo, las futuras versiones de MultiGWAS deberían incluir ploidías más complejas que los tetraploides y el cálculo explícito de parámetros, ya sea para filtrar los datos poliploides antes del análisis GWAS o para complementar los parámetros de otras poblaciones genómicas de los datos analizados.

## 7 Agradecimientos

Esta investigación fue posible gracias al macroproyecto de AGROSAVIA titulado “Investigación en conservación, caracterización y uso de los recursos genéticos vegetales” que tuvo cinco años de duración.

Agradecemos al Ministerio de Ciencia, Tecnología e Innovación de la República de Colombia (antes COLCIENCIAS), por el apoyo al investigador postdoctoral LG en AGROSAVIA durante 2019-2020 bajo la supervisión de ICS y PHRH (Beca número 811-2019). La editorial de AGROSAVIA dio para el aval para publicar este estudio. Finalmente, gracias a Andrés J. Cortés por sus valiosos comentarios para mejorar este manuscrito.

## 8 Contribuciones de los autores

LG, ICS y PHRH concibieron la idea. LG desarrolló MultiGWAS. MP probó MultiGWAS. Todos los autores escribieron y aprobaron la versión final del manuscrito.
